# Experimental Study of Emulative Precast Concrete Beam-to-Column Connections Locally Reinforced by U-Shaped UHPC Shells

**DOI:** 10.3390/ma15124066

**Published:** 2022-06-08

**Authors:** Lei Tang, Wenhua Tian, Dongzhi Guan, Zixuan Chen

**Affiliations:** 1 School of Civil Engineering, Southeast University, Nanjing 211189, China; seutanglei@163.com (L.T.); twh9809@163.com (W.T.); chenzixuan94@163.com (Z.C.); 2 Key Laboratory of Concrete and Prestressed Concrete Structure of Ministry of Education, Southeast University, Nanjing 211189, China

**Keywords:** precast concrete, UHPC shell, beam-to-column connection, cyclic loading, seismic behavior

## Abstract

Precast beam–column connections act as vital elements of precast concrete frames. To enhance the resistance to the earthquake-induced damage and environment-induced deterioration of precast beam–column connections, an innovative precast concrete beam-to-column connection locally enhanced by prefabricated ultra-high-performance concrete (UHPC) shells was proposed. For studying the seismic behaviors of these novel connections and the influence caused by the prefabricated UHPC shell length, full-scale precast specimens were experimentally investigated using low-cyclic reversed loading tests. The obtained results were analyzed and discussed, including hysteresis curves, skeleton curves, strength and deformability, performance degradation, energy dissipation capacities, and plastic hinge length. The results reveal that the novel precast concrete beam–column connections with UHPC shells behaved satisfactorily under seismic loadings. The damage in the concrete near the lower part of the beam end is reduced by the prefabricated UHPC shells. The longer prefabricated UHPC shells were more useful for decreasing the damage to the precast concrete components and improved the structural performance. The precast specimen with 600-mm long UHPC shells can achieve a ductility of 4.87 and 4.0% higher strength than the monolithic reference specimen.

## 1. Introduction

Buildings constructed with prefabricated concrete elements have been deemed a basic construction method since the invention of normal concrete and they remain increasingly popular in the construction industries of the current world. In China, as the construction industry expands and green development is being promoted, structures with prefabricated concrete components have become the necessary choice for new concrete buildings in many areas due to their inherent advantages, such as high production efficiency, short construction period, good quality, and low environmental pollution [1]. Seismic performance is important for building structures in earthquake areas [2]. Compared with the seismic performance of monolithic concrete structures, precast concrete structures are classified into two types, emulative systems and independent systems [3]. Emulative precast concrete systems require that the seismic performance and even structural details of precast concrete structures remain identical to—or even slightly higher and stronger than—those of monolithic concrete structures, so that designers and contractors are able to apply precast concrete structures easily and quickly. Thus, emulative precast concrete structures are still the main choice of large-scale-applied precast systems around the world.

In precast concrete structures, the connections between different prefabricated elements are the inherent positions where the stiffness of components changes. Under earthquake attacks, the seismic loading-induced damage in precast concrete structures is prone to concentrate at the connection zones locally [4,5,6,7], leading to relatively smaller damaged areas than those in monolithic structures [8,9]. The energy dissipation capacity of emulative precast structures, relying on the crushing of concrete and the yielding of reinforcements, is generally slightly inferior to that of monolithic concrete structures with the same basic configurations and reinforcements. In practice, either the longitudinal reinforcements in the precast components [8] or the local stirrups at the connection zones [10,11] are increased to ensure the design philosophy of the precast emulative system—e.g., the longitudinal reinforcing rebars of the precast beam–column connection in [8] are actually improved, including both the beam bottom longitudinal rebars and the lap-splicing rebars, and the stirrups are spaced at small intervals at the both sides of the precast concrete walls in [10]. However, the improvement of reinforcements and stirrups in precast structures results in difficulties in manufacturing and construction at sites, causing adverse impacts on the construction efficiency and cost of precast structures.

Beginning development in the early 1960s, ultra-high-performance concrete (UHPC) is a new cementitious material generally consisting of high-content binder, fine aggregates, superplasticizers, and low water–binder ratio (0.2–0.3). Compared with conventional concrete, UHPC demonstrates ultra-high-performance, including strength (compressive strength > 100, 120 or 150 MPa), workability (>260 mm), superior durability, etc. [12,13]. Therefore, it has gradually been regarded as a basic building material with great promise. To increase the resistance to earthquake-induced damage and environment-induced deterioration of emulative precast systems without reducing the advantages of precast concrete structures, UHPC is increasingly employed at the connection zones between precast elements for its excellent strength, ductility, and durability [14,15,16,17], especially in precast beam–column connections. Maya et al. [18] tested four full-scaled precast beam–column connection specimens under reversal cyclic loadings, of which the reinforcing bars at the beams were lap-spliced in UHPC at precast beam ends. On the other hand, Xue et al. [19] poured UHPC into the beam–column joint core as the lower portion to embed the bottom longitudinal rebars of precast concrete beams. Experimental investigations showed that the prefabricated concrete beam-to-column connections behaved well under the cyclic loadings in the condition of the reduced anchoring length of the beam bottom reinforcing bars. Zhang et al. [20], Ma et al. [21], and Ma et al. [22] replaced the entire normal concrete in the precast beam-to-column joint core by post-pouring UHPC, and acceptable seismic behaviors of these connections were proven in reversal cyclic loading tests. Lin et al. [23] studied newly proposed prefabricated beam-to-column connections using prefabricated UHPC shells at beam-to-column joint cores for both enhancing the joint and acting as the formwork. It is demonstrated that most investigations focused on the emulative prefabricated beam-to-column connections using post-pouring UHPC at the connection zones for simplifying connecting details or improving seismic performance and durability. However, due to the high requirements of pouring and curing UHPC, it is inefficient and difficult for contractors to pour a small amount of UHPC when a relatively large quantity of normal concrete is poured.

In this investigation, an innovative beam-to-column connection in an emulative prefabricated concrete frame—which is locally reinforced by U-shaped prefabricated UHPC shells—was proposed to enhance the resistance to the earthquake-induced damage and environment-induced deterioration without increasing the construction costs and difficulties at sites. The UHPC shell is prefabricated at factories together with the precast concrete beams, the reinforcing rebars and construction process remain identical to that of conventional precast concrete structures that are familiar to contractors. An experimental study was conducted on full-scaled specimens to investigate the seismic behaviors of this innovative connection.

## 2. Structural Details of the Innovative Connection

Precast beam ends connected to columns of emulative precast concrete frames in China are usually designed to bear the so-called negative moments, putting the upper fiber of beam ends in tension and the bottom fiber in compression. Therefore, the ratio of the upper longitudinal reinforcing bars at the beam end becomes commonly higher than that of the lower rebars. When cyclic loadings induced by earthquakes occur, the lower concrete at the beam ends is often severely damaged, which is prone to become the controlling factor determining the overall seismic behaviors of the beam-to-column connection following the structural design philosophy of a “strong column–weak beam”. This is proved by numerous investigations [5,8,11]. Simultaneously, considering the high requirements and low efficiency of pouring and curing UHPC into precast concrete structures, it is more suitable to prefabricate UHPC elements for their application in precast concrete structures.

**Figure 1:**
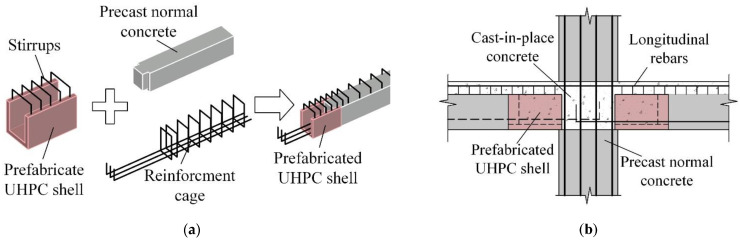
Schematic of the novel precast beam-to-column connection. (**a**) Precast beam, (**b**) connection.

In this study, based on the widely used precast configurations, a prefabricated UHPC shell is added to make U-shaped hollow sections at the beam end in a manufacturing factory, strengthening the bottom portion at the precast concrete beam ends, as shown in Figure 1. Some normal concrete is set into the prefabricated UHPC shell with a certain length to connect them as one precast element. Near the beam end regions, the bottom longitudinal reinforcing bars extend from the conventional concrete portion through the hollow portion formed by the UHPC shell. The stirrups at the beam end region are set into the prefabricated UHPC shell. The on-site installation process of the novel beam–column connection is completely identical to that of normal emulative precast concrete frame—i.e., (a) erecting the precast concrete columns, (b) installing the precast beams making the beam bottom longitudinal rebars extrude into the beam–column joint cores, and (c) pouring normal concrete to connect all the precast elements. The beam ends of the novel beam–column connections become UHPC-normal concrete composite elements, which are expected to transfer the damaged areas at the beam end bottom away with improved seismic performance. Compared with the entire frame structure, UHPC accounts for a very small percentage, which scarcely affects the construction cost. The construction process of the innovative precast beam–column connections remains easy for contractors to accept and can be widely applied.

## 3. Experimental Investigation

### 3.1. Specimen Description

**Figure 2:**
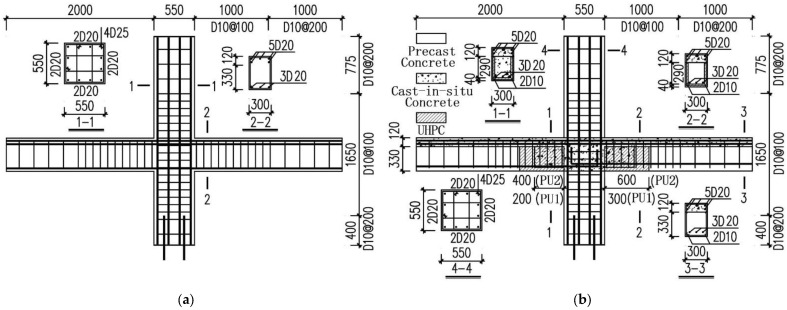
Configurations and reinforcements of the specimens. (**a**) Specimen S1, (**b**) Specimens PU1 and PU2.

A high-rise building comprised of precast frames and monolithic shear walls in China was selected as prototype structure, based on which the test specimens were designed. Figure 2 depicts the configurations and reinforcements of the specimens. The specimen labeled S1 in another study was adopted as the monolithic reference specimen in this investigation [8]. Two precast specimens with U-shaped UHPC shells were designed to have the same configurations and reinforcement assignments as Specimen S1, which were designated PU1 and PU2, respectively. Precast multistory columns were adopted in the precast specimens with a 550 × 550 mm cross-section, indicating that the longitudinal rebar went continuously throughout the entire height of the specimens. The height of the monolithic layers above the precast beams, of which the sectional height was 330 mm, remained 120 mm. The bottom longitudinal rebars with upwards end-hooks in the precast beams were anchored inside the joint cores, of which the anchorage length was 450 mm. The stirrups of the beams near the joint and the lateral ties of the columns were placed with 100 mm intervals to enhance the connection regions according to the Chinese design code [24]. The 300 mm and 600 mm long prefabricated UHPC shells with a 40-mm thickness were set in Specimens PU1 and PU2, respectively. The U-shaped hollows at the beams formed by the UHPC shells were 200 mm and 400 mm, respectively.

**Figure 3:**
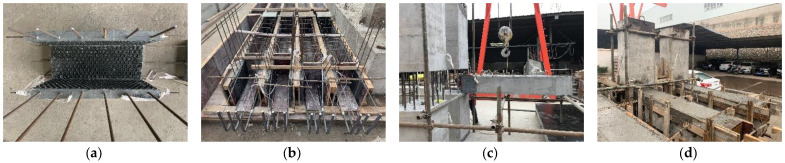
Manufacturing of the precast specimens. (**a**) Making UHPC shells, (**b**) fabricating beams, (**c**) installing beams, (**d**) pouring concrete.

Figure 3 shows the manufacturing process of the two precast specimens. Due to the complicated configurations of the precast specimens, the wood formwork is utilized for the production. The prefabricated UHPC shells were made in an inverted position for easy fabrication. First, 25-mm-diameter bubble wraps were pasted onto the inner formwork to increase the roughness of the inner interfacial surface of the prefabricated UHPC shells that contacted the normal monolithic concrete [15]. The stirrups were mounted with U-shaped configurations to facilitate making other formworks. After the pouring of UHPC, the curing agent was sprayed on the surface, helping the curing process. The precast beams were manufactured following the steps: (a) making the rebar cages, (b) placing the UHPC shells and the rebar cages in the formworks, (c) pouring and curing the normal concrete, and (d) bending the straight rebars at the prefabricated UHPC shells to form the whole stirrups. During the manufacturing process, the conventional concrete was made and poured in an ordinary manner, and hand-held concrete vibrators were used for vibrating and compacting the conventional concrete. The upper side surfaces of the concrete were finished manually. The conventional concrete was cured in an outdoor environment and covered with the protective plastic films. After the beams hardened, they were lifted and mounted beside the prefabricated columns. After pouring and curing normal concrete at the monolithic layers and the connection regions, the precast specimens were finished.

### 3.2. Material Properties

A local commercial company in Jiangsu, China offered the proprietary UHPC material used for these specimens. The raw materials for the UHPC consisted of reactive powder, sand, water, steel fibers, and admixtures, of which the proportions are listed in Table 1. The reactive powder was produced by the provider in advance by mixing cement and highly active multicomponent admixtures, of which the apparent density was 2.94 g/cm^3^ [25]. Moreover, the residual volume ratios of the reactive powder through 0.08 µm and 0.045 µm sized sieves were 18% and 27%. 

Normal concrete of Grade C40 and reinforcements of Grade HRB400 were adopted to make the test specimens. The control cubes of two different sizes were cast to obtain the compressive strength of UHPC and normal concrete [24,26], respectively. The tensile strength of UHPC was gained from the dog-bone-shaped specimens [27]. These specimens for material properties were manufactured at the same time and cured in the same surroundings as the beam–column specimens. All the coupon tests were carried out just before the beam–column specimens were loaded cyclically. Table 2 and Table 3 summarizes the material properties of the employed concrete and rebars in the precast specimens, respectively. The material properties of Specimen S1 are presented in [8].

### 3.3. Test Setup and Loading Protocol

**Figure 4:**
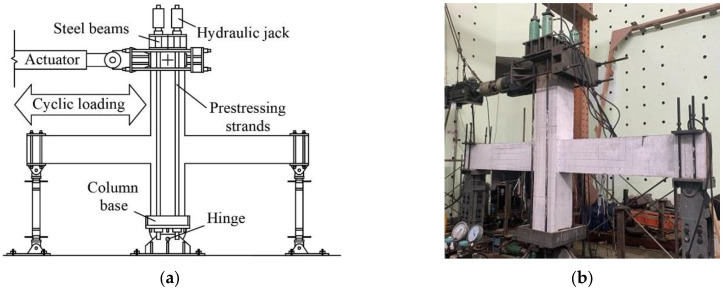
Test setup. (**a**) Schematic, (**b**) pictures.

To simulate the deformation mode of an idealized frame in which the moment at the midspan of the column and the beams approached zero, the test setup illustrated in Figure 4 was employed. The test specimen was mounted onto a hinged base with the beam ends fixed on sliding roller supports. The lateral reversal cyclic loadings were applied to the upper column end through the loading head connected to a 1000-kN MTS actuator. Four hydraulic jacks were employed to simulate the vertical loadings based on the prototype structure, which were connected to the column base through four bundles of prestressing strands. The vertical distance between the hinges of the column was determined as 2900 mm, the hinges of the beams were set 4250 mm far away from each other due to the restraints in the laboratory and the characteristics of the prototype structure.

**Figure 5:**
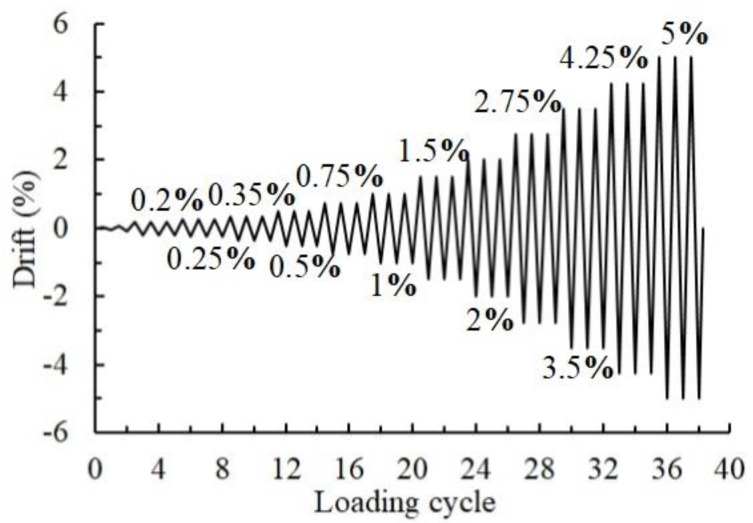
Loading sequence [9].

Based on the load-bearing conditions of the high-rise prototype structure, the vertical column axial load ratio remained equal to 0.22. As shown in Figure 5, a displacement-controlled lateral loading sequence was employed in this test according to ACI 374.1-05 [28]. The drift was calculated by dividing the actuator-applying loading displacement to the distance between the upper and bottom hinged supports connected to the column. For checking the function of the loading system, preloadings of 2 mm and 4 mm were applied once before the formal loadings. A total of 12 loading levels—of which each was loaded three times—were planned to be applied to the specimens, i.e., 0.2%, 0.25%, 0.35%, 0.5%, 0.75%, 1%, 1.5%, 2%, 2.75%, 3.5%, 4.25%, and 5%. The loading procedure was terminated when the peak strength of a loading cycle dropped by 20% of the maximum strength.

## 4. Results and Discussions

### 4.1. Damage and Failure Modes

**Figure 6:**
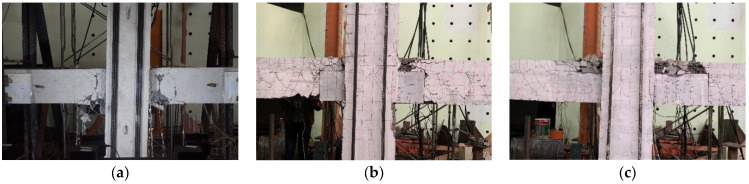
Failure modes of specimens. (**a**) S1, (**b**) PU1, (**c**) PU2.

As presented in Figure 6, it was obvious that all the specimens failed to show the beam sideway mechanism, of which the main damage appeared at the beam ends with minor damage at the columns. This proved that the adopted design principle of “strong column–weak beam” was valid for the novel beam-to-column connections with prefabricated UHPC shells. For Specimen S1, the lower portion at the beam ends was more severely damaged than the upper portion because the bottom longitudinal rebars were less. Main wide cracks were observed at the beam ends, which were approximately 10 cm away from the beam-to-column interface. The precast specimens showed different damage characteristics from the monolithic reference specimen. The prefabricated UHPC shells in the precast specimens effectively mitigated the damage to the bottom portions of the beam ends. However, it was interesting that the damage to the monolithic layers on the precast beams of Specimens PU1 and PU2 seemed more severe than that found on the corresponding region of Specimen S1. For Specimen PU1, there was a small amount of concrete crushed at the beam bottom near the joint location where the normal concrete contacted the UHPC shells, resulting in the bottom longitudinal rebars also being bent under compression. The UHPC shell of Specimen PU2 was expected to be longer than the predicted plastic hinge. Some upwards vertical fine cracks were observed in the UHPC shells, indicating that the UHPC shells—especially the bottom portions—bore considerable tension during the loading process. No spalling of concrete appeared at the beam bottom, while the monolithic concrete layers on the beams crushed substantially. For the failure modes of the specimens adopting UHPC at the connection regions in [18,20,23], the beam ends were damaged at both the bottom and upper areas under reversal cyclic loadings while the bottom areas of the beam ends in the proposed connections were protected well by the prefabricated UHPC shells with minor or even negligible damage.

### 4.2. Hysteresis and Skeleton Curves

**Figure 7:**
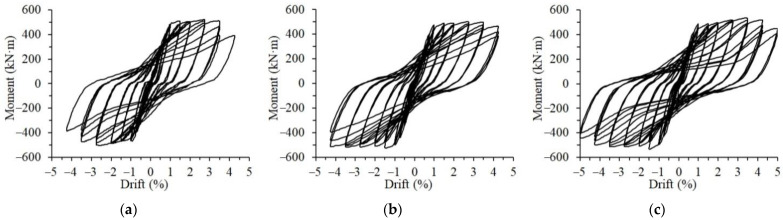
Hysteresis curves of the test specimens. (**a**) S1, (**b**) PU1, (**c**) PU2.

Figure 7 presents the moment–drift relationships of the three test specimens. The overall shapes of the hysteresis curves of all the specimens were stable and plump, indicating good seismic energy dissipation capability. At loading levels of 0–2.75%, the three hysteresis loops at one loading level were quite close because the damage of each specimen was minor. When the loading drift reached 3.5%, some concrete in the test specimens began to spall, the hysteresis loops started to show some pinching effect of different degrees consequently. The pinching effect of the hysteresis curves of Specimens S1 and PU2 seemed more severe than that of Specimen PU1. When the 4.25% loading drift amplitude was applied, Specimen S1 was destroyed due to the damage of the beam ends, showing that the longitudinal rebars at the beam bottom bent under compression. The corresponding hysteresis curve became an S-shaped loop, and the peak load dropped by 20% of the ultimate load during the entire loading process, which was regarded as being destroyed. Specimen PU1 underwent three loading cycles at the 4.25% loading level before being destroyed. For Specimen PU2, the strength was maintained even though the hysteresis loops at loading levels higher than 3.5% became quite narrow. The loading test on Specimen PU2 was terminated after two loading cycles of 5% loading amplitude because of the considerable drop in strength.

**Figure 8:**
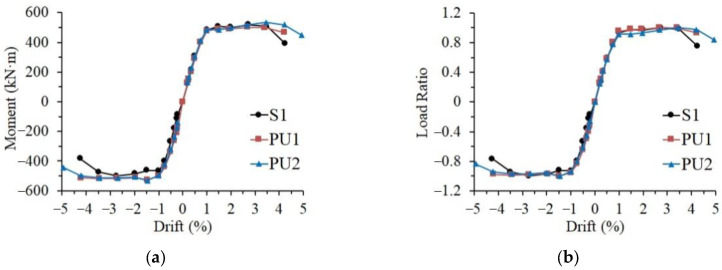
Skeleton curves of the test specimens. (**a**) Actual curve, (**b**) normalized curve.

By picking up the peak points in the hysteresis curves at different loading amplitudes, the actual skeleton curves were obtained, as shown in Figure 8a. The precast specimens were manufactured from the concrete and the reinforcing rebars of the same grade as the monolithic reference specimens at different times. To reduce the influence of the minor difference between the strength of the employed materials at different times, the positive and negative applied loads of the three specimens were normalized, being divided by the corresponding maximum strength that each specimen achieved. Figure 8b presents the normalized skeleton curves of all the specimens that were relatively close, starting to yield at approximately the 1% loading drift. When approaching failure, the strength of the monolithic reference specimen decreased significantly, while the skeleton curves of the precast specimen remained relatively stable. This indicated that the UHPC shells helped to maintain the stable structural performance of the precast specimens. Specimen PU2 with longer UHPC shells showed the best deformation capacity, reaching a loading drift of 5%.

### 4.3. Performance of Strength and Deformability

The strength and deformability are important indices to compare the seismic performance of the monolithic and precast specimens. The turning points of the skeleton curves were considered the yield points of the specimens, of which the corresponding moment and loading drift were adopted as the yield moment and yield drift. The critical strength of the three specimens is shown in Table 4. As shown in Table 4, the strength of Specimen PU1 was comparable to that of Specimen S1, while the maximum strength of Specimen PU2 was 4.0% higher than that of Specimen S1 in average. Regarding the average values of the ratio calculated by dividing the maximum strength by the yield strength, a similar conclusion could be reached. This indicated that the longer UHPC shells could be conducive to the load-bearing capacity and the safety assurance in strength.

Ductility was utilized to evaluate deformability, which was calculated by dividing the ultimate drift by the yield drift. The ultimate drift was gained as the corresponding loading drift when the applied load fell to 80% of the maximum load. As listed in Table 5, the specimens achieved ultimate drifts larger than 4%, indicating the collapse prevention (CP) performance level [29]. The ductility values of Specimens S1, PU1, and PU2 were at 3.82, 3.95, and 4.87, respectively, showing considerable deformation capacity. The precast specimens with the UHPC shells exhibited higher deformability than the monolithic reference specimen. Specimen PU2 achieved the highest ductility value, which was also higher than the specimens in [20,23]. This indicated that the long UHPC shells could improve the deformability of the beam–column connections significantly.

### 4.4. Performance Degradation

**Figure 9:**
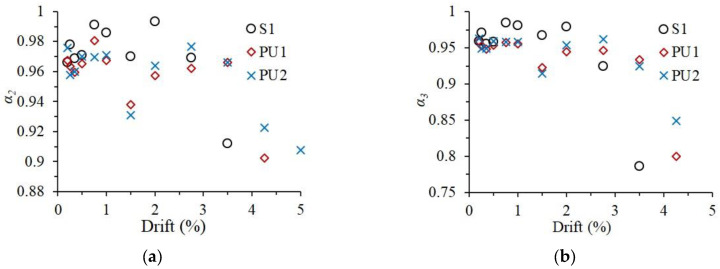
Strength coefficient of the three specimens. (**a**) *α_2_*_,_ (**b**) *α_3_*.

Due to the accumulation of damage, the structural performance of the specimens deteriorates with increasing applied cyclic loadings. Strength and stiffness degradation are always utilized to evaluate seismic performance degradation. As defined in Equation (1), the coefficient of strength, employed for strength deterioration, is calculated as the result dividing the peak load in the second or third loading cycle at a certain loading level by the corresponding first peak load.

(1) $$\alpha_{i}=P_{i}^{j}/P_{1}^{j}$$

where $\alpha_{i}$ is the coefficient of strength, $P_{1}^{j}$ and $P_{i}^{j}$ are the peak load in the first and *i*-th loading cycle at the *j*-drift loading level and *i* equals 2 or 3. The average values of the strength coefficients in the positive and negative directions are compared in Figure 9. At loading levels below 2% drift, which was considered the life safety performance level [29], the strength coefficient of Specimen S1 was higher than those of Specimens PU1 and PU2, indicating the lower strength degradation of the monolithic specimen. However, with increasing loading levels, the strength coefficient of Specimen S1 dropped significantly, becoming smaller than those of Specimens PU1 and PU2. This demonstrated that the UHPC shells were helpful for the strength stability of the precast specimens at large loading drifts. Based on the values of the strength coefficient, the longer UHPC shell was more favorable for reducing the strength degradation of the precast specimen. The $\alpha_{3}$ values of Specimens PU1 and PU2 at the loading drift of 3.5% were 0.934 and 0.925, respectively, which conformed to the requirement of exceeding 0.75 in ACI 374.1-05 [28].

**Figure 10:**
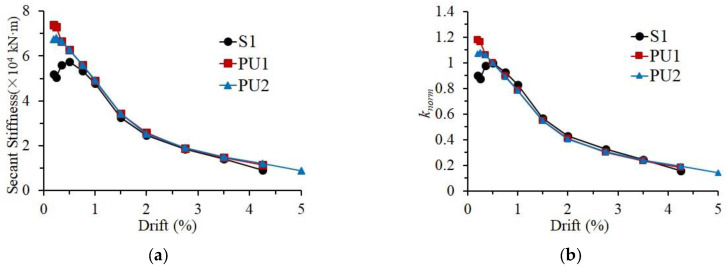
Stiffness degradation of the specimens. (**a**) Actual curve, (**b**) normalized curve.

Regarding the stiffness deterioration, the secant stiffness—defined in Equation (2)—of the first loading cycle at certain loading level is utilized to characterize the stiffness degradation of the specimens.

(2) $$K_{j}=(\left|+P_{1}^{j}\right|+\left|-P_{1}^{j}\right|)/(\left|+d_{1}^{j}\right|+\left|-d_{1}^{j}\right|)$$

where $+P_{1}^{j}$ and $-P_{1}^{j}$ are the positive peak loads and the negative peak loads in the first loading cycle at *j*-drift loading level, $+d_{1}^{j}$ and $-d_{1}^{j}$ are the corresponding loading drifts, respectively. As illustrated in Figure 10a, at the initial loading stage (below 0.5% loading drift), some slips in the test setup occurred during the loading process of Specimen S1, leading to the relatively low stiffness of Specimen S1. After that, the slips were eliminated, and the stiffness values beyond the 0.5% loading drift were believed to be reliable. In general, the stiffnesses of the three specimens beyond the loading level of 0.5% drift were quite close. For a better comparison of stiffness degradation, the normalized stiffness, *k_norm_*, was calculated as the ratio of the secant stiffness to the values corresponding to the 0.5% loading drift. The normalized stiffness curves are presented in Figure 10b. Owing to inherent assembly gaps between the precast elements, the stiffness degradation of precast specimens during the loading amplitude below the 2.75% loading drift was more serious than that of Specimen S1. When the specimens approached failure, the monolithic reference specimen deteriorated in stiffness more severely than the precast specimens, revealing that the UHPC shells could help the beam–column specimens ameliorate the stiffness degradation, especially at the large loading drifts.

### 4.5. Capacity of Energy Dissipation

**Figure 11:**
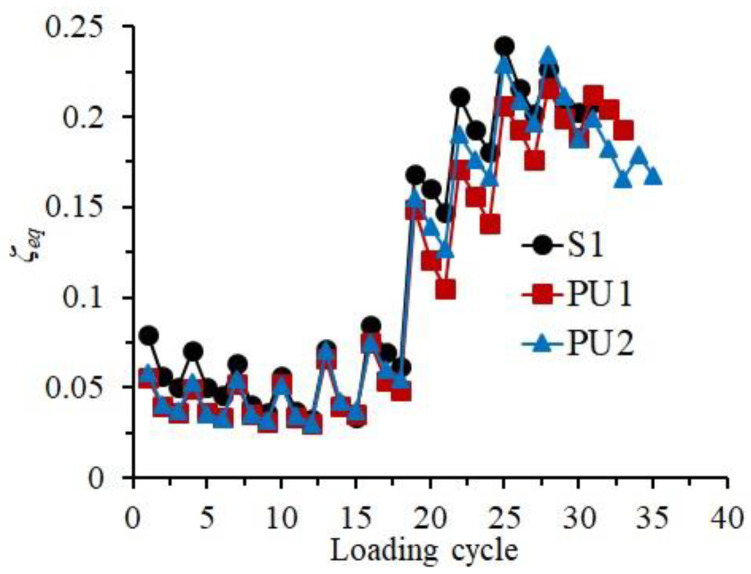
Equivalent viscous damping ratio of the specimens.

As one of the most important indicators of seismic performance, the capacity of energy dissipation of structures or components is usually evaluated by two different indices, the equivalent viscous damping ratio (*ζ_eq_*) and cumulative dissipated energy. As the test specimens in this paper were manufactured at different times, the values of *ζ_eq_* were calculated and utilized in this investigation, which equaled the result of dividing the dissipated energy in one hysteresis loop by the product of the dissipated energy in an equivalent linear system and a constant, 2π [23]. Figure 11 exhibits the relationship between the *ζ_eq_* values of the three specimens and the number of loading cycles. The variation characteristics of the presented curves of the three specimens were relatively close, showing a similar developing process of the energy dissipation capacity as the loading process. Except for the failure stage, the equivalent viscous damping ratio of Specimen S1, due to the better integrity, was marginally higher than those of Specimens PU1 and PU2, showing a relatively better capacity of energy dissipation. In the failure stage, the *ζ_eq_* values of Specimens PU1 and PU2 became comparable with that of Specimen S1, and the total number of loading cycles was also larger than that of Specimen S1. This revealed that the prefabricated UHPC shells were able to increase the energy dissipation capacity of the precast specimens at the failure stage. The *ζ_eq_* values of Specimen PU2 remained greater than those of Specimen PU1 in the main loading stage, indicating that the longer UHPC shell was more helpful for improving the energy dissipation capacity.

### 4.6. Length of Plastic Hinges

**Figure 12:**
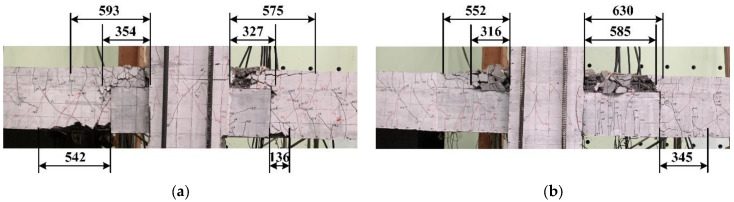
Length of plastic hinges of the specimens (mm). (**a**) Specimen PU1, (**b**) Specimen PU2.

For a reinforced concrete member, the plastic hinge not only affects the plastic deformation capacity but also influences the damaged regions of the member under reversed cyclic loadings. There are two concepts of plastic hinge length, of which one is that the length of a plastic hinge is an assumed value related to the calculation of a concrete member deformation. The other concept proposed by investigators is that the length of a plastic hinge is a real physical parameter related to concrete damage [30,31], and can be measured [32]. In this paper, the length of the crushed-concrete area at the beam end was considered to be related to the length of a plastic hinge. Owing to the characteristics of the novel connection, the length of the crushed concrete at the upper and lower portions of the monolithic layers on the beams and at the bottom of the beams was measured, respectively, as exhibited in Figure 12. The mean value of the three measured lengths was considered to be the equivalent plastic hinge length of one beam, and the average value obtained from the equivalent plastic hinge length of the two beam ends in one specimen was regarded the representative length of plastic hinges (*L_p_*). As summarized in Table 6, the representative lengths of plastic hinges of the two precast specimens were quite close. By comparison, Specimen PU2 owned a slightly smaller length of plastic hinges, indicating that the longer prefabricated UHPC shell could effectively reduce the concrete-damaged region of the novel connection and improve the overall performance.

## 5. Conclusions

An innovative beam-to-column connection in an emulative precast concrete frame was put forward, which was locally enhanced by U-shaped UHPC shells. The seismic behaviors of the innovative beam–column connections were studied by reversal cyclic loading tests on the cruciform full-sized specimens. Conclusions can be reached based on the experimental investigations, as follows:

(1) The precast concrete beam–column connections, locally reinforced by U-shaped UHPC shells, exhibited good seismic performance with comparable hysteresis behaviors that were close to the ones of the monolithic reference specimen.
(2) The failure mode of the beam sideway mechanism was achieved by the novel connections; thus, the principle of a “strong column–weak beam” could be used to design the proposed connection.
(3) In comparison with the monolithic reference specimen, the prefabricated UHPC shells could reduce the damage in the concrete near the lower parts of the beam ends. They could also enhance the seismic behaviors of the precast concrete specimens under large loading drifts and maintained better capacity of load-bearing and energy dissipation when the precast specimens approached failure.
(4) The longer precast UHPC shell was more conducive to reducing the concrete damage of the precast specimens and improving the strength, ultimate deformation capacity, and capacity of energy dissipation. The precast specimen with 600-mm long UHPC shells can achieve a ductility of 4.87 and 4.0% higher strength than the monolithic reference specimen.

Note: The analysis and conclusions presented in this paper are restricted to the specimens designed according to the principle of “strong column–weak beam”.

## Figures and Tables

**Table 1:** Mixture proportions of the UHPC used (kg/m^3^).

Reactive Powder	River Sand ^a^	Water	Steel Fibre ^b^	Admixture ^c^
1170	930	182	160	22

Notes: ^a^ The density of the river sand was 2.82 g/cm^3^; ^b^ The length, diameter, and tensile strength of the copper plated steel fibers were 13 mm, 0.2 mm, and 2800 MPa, respectively; ^c^ The water reduction rate of the polycarboxylate high-performance water reducer was more than 30%.

**Table 2:** Concrete material properties.

Specimen	Cubic Compressive Strength (MPa)	Tensile Strength (MPa)
UHPC	103.7	7.82
Concrete of precast elements	40.7	-
Cast-in-place concrete	41.3	-

**Table 3:** Reinforcing rebar material properties.

Reinforcing Rebars	Diameter (mm)	Yield Strength (MPa)	Ultimate Strength (MPa)
Longitudinal rebars	20	413	580
	25	439	598
Stirrups	8	613	710
	10	542	635

**Table 4:** Comparison of the strength of specimens.

Specimen	Direction	Yield Strength (kN·m)	Maximum Strength (kN·m)	Ratio of Maximum Strength to Yield Strength	Average
S1	Positive	484.5	520.8	1.08	1.075
	Negative	−467.8	−501.9	1.07	
PU1	Positive	462.7	500.9	1.08	1.070
	Negative	−496.8	−526.5	1.06	
PU2	Positive	485.4	533.5	1.10	1.085
	Negative	−496.2	−530.3	1.07	

**Table 5:** Comparison of the ductility of specimens.

Specimen	Direction	Yield Drift	Ultimate Drift	Ductility	Average
S1	Positive	1.00	4.07	4.08	3.82
	Negative	−1.12	−4.00	3.57	
PU1	Positive	1.14	4.23	3.71	3.95
	Negative	−1.00	−4.18	4.18	
PU2	Positive	0.99	4.98	5.03	4.87
	Negative	−1.06	−4.98	4.70	

**Table 6:** Length of plastic hinges of specimens (mm).

Specimens	Left				Right				*L_P_*
	Top	Middle	Bottom	Average	Top	Middle	Bottom	Average	
PU1	593	354	542	496	575	327	136	346	421
PU2	552	316	0	289	630	585	345	520	405

Note: *L_P_* is the representative length of plastic hinges of the test specimen.

## Data Availability

Not applicable.
